# Endoscopic drainage and necrosectomy of a biloma facilitated by endoscopic ultrasound-guided gastro-gastrostomy

**DOI:** 10.1055/a-2686-2631

**Published:** 2025-09-09

**Authors:** Jantien A. Vogel, Rutger-Jan Swijnenburg, Rogier P. Voermans, Mattheus C. B. Wielenga, Roy L. J. van Wanrooij

**Affiliations:** 1 26066 Department of Gastroenterology and Hepatology, Amsterdam UMC, University of Amsterdam, Amsterdam, The Netherlands; 2 1209 Department of Surgery, Amsterdam UMC, University of Amsterdam, Amsterdam, The Netherlands; 3 1209 Department of Gastroenterology and Hepatology, Amsterdam UMC, Vrije Universiteit, Amsterdam, The Netherlands

A 28-year-old female with a history of Roux-en-Y gastric bypass and colorectal liver metastases underwent combined metastectomy and microwave ablation of segments 1, 2, 3, and 8. A postprocedural biloma was drained with a percutaneous drain, while percutaneous transhepatic drain placement failed to treat the biliary leak. An EUS-guided gastro-gastrostomy was created using a 20-mm cautery-enhanced lumen-apposing metal stent (LAMS) to facilitate endoscopic retrograde cholangiopancreaticography (ERCP) with placement of biliary plastic stents ( Video 1 ). Both the percutaneous drain and the biliary stents clogged repeatedly due to the large amount of necrotic contents, resulting in multiple infectious episodes. EUS-guided drainage of the biloma was subsequently performed from the excluded stomach using a 15 mm × 10 mm LAMS ( Fig. 1 ). A double pigtail stent was placed through the LAMS to prevent stent migration and dysfunction. The patient initially recovered but after 10 days again developed stent dysfunction. During three endoscopic necrosectomy sessions, all necrotic tissue in the biloma was removed using a snare and grasping forceps ( Fig. 2 ). The LAMS was then exchanged for two plastic pigtails, and both the percutaneous drain and biliary stents were removed. The patient recovered well, and a CT scan 3 months post-drainage showed only a small biloma remnant.

Endoscopic drainage and necrosectomy of a necrotizing biloma facilitated by endoscopic ultrasound-guided gastro-gastrostomy.Video 1

**Fig. 1:**
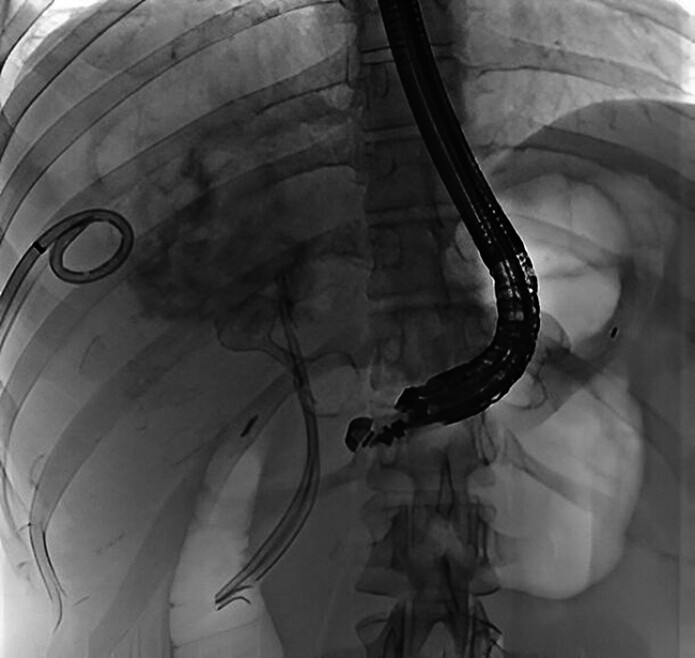
EUS-guided drainage of biloma from the excluded stomach using a 15-mm lumen-apposing metal stent.

**Fig. 2:**
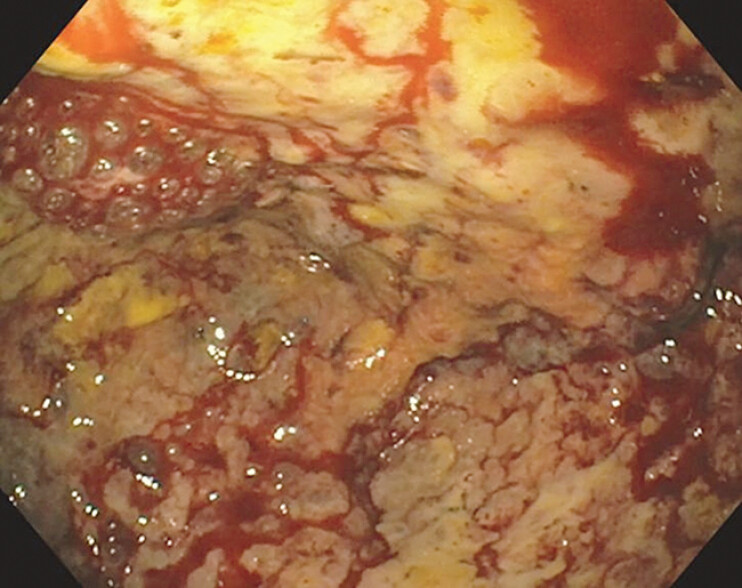
Endoscopic view of the biloma cavity after necrosectomy.

In selected cases where standard therapy for symptomatic biloma – percutaneous drain – fails, EUS-guided transluminal biloma drainage may be performed, which in this patient offered several benefits. First, the challenges of surgically altered anatomy were overcome by EUS-guided gastro-gastrostomy, facilitating transgastric ERCP (EDGE) and other endoscopic interventions (EDGI) 1 . Second, internal drainage of the biloma precluded the need for a percutaneous drain and improved the patient’s comfort 2 . Third, placement of a large caliber LAMS enabled necrosectomy that initiated clinical recovery. Endoscopic necrosectomy has previously been shown to be effective in treating necrotic peripancreatic collections and gangrenous cholecystitis, with its potential applications continuing to expand 3 4 .

Endoscopy_UCTN_Code_TTT_1AS_2AJ

## Notes

Endoscopy E-Videos https://eref.thieme.de/e-videos
*E-Videos* is an open access online section of the journal *Endoscopy* , reporting on interesting cases and new techniques in gastroenterological endoscopy. All papers include a high-quality video and are published with a Creative Commons CC-BY license. Endoscopy E-Videos qualify for HINARI discounts and waivers and eligibility is automatically checked during the submission process. We grant 100% waivers to articles whose corresponding authors are based in Group A countries and 50% waivers to those who are based in Group B countries as classified by Research4Life (see: https://www.research4life.org/access/eligibility/ ). This section has its own submission website at https://mc.manuscriptcentral.com/e-videos .
